# Supplementary material for: HIVprotI: an integrated web based platform for prediction and design of HIV proteins inhibitors
Source: J Cheminform. 2018 Mar 9;10:12. doi: 10.1186/s13321-018-0266-y (PMC5845081; doi:10.1186/s13321-018-0266-y)
Supplement: Supplementary file 2 — Additional file 2. Source code of HIVProtI web server. [file 13321_2018_266_MOESM2_ESM.zip › HIVprotI_Source-code/hivprotI/weka-3-6-6/doc/weka/gui/beans/CustomizerCloseRequester.html]

CustomizerCloseRequester


---


|  |  |  |
| --- | --- | --- |
| **Overview** \| **Package** \| **Class** \| **Tree** \| **Deprecated** \| **Index** \| **Help** \| **Weka's home** | |  |
| **PREV CLASS**   **NEXT CLASS** | **FRAMES**    **NO FRAMES**     **All Classes** |  |
| SUMMARY: NESTED \| FIELD \| CONSTR \| METHOD | DETAIL: FIELD \| CONSTR \| METHOD |  |


---


## weka.gui.beans Interface CustomizerCloseRequester

**All Known Implementing Classes:**: AssociatorCustomizer, ClassAssignerCustomizer, ClassifierCustomizer, ClassValuePickerCustomizer, ClustererCustomizer, FilterCustomizer, LoaderCustomizer, SaverCustomizer, SerializedModelSaverCustomizer

---

``` public interface CustomizerCloseRequester ```

Customizers who want to be able to close the customizer window
themselves can implement this window. The KnowledgeFlow will
pass in the reference to the parent JFrame when constructing
the customizer. The customizer can then call dispose() the
Frame whenever it suits them.

**Version:**
:   $Revision: 1.2 $

**Author:**
:   Mark Hall

---

| **Method Summary** | |
| --- | --- |
| `void` | `setParentFrame(javax.swing.JFrame parent)`             A reference to the parent is passed in |

**Method Detail**

### setParentFrame

```
void setParentFrame(javax.swing.JFrame parent)
```

:   A reference to the parent is passed in

    :   **Parameters:**: `parent` - the parent frame


---


|  |  |  |
| --- | --- | --- |
| **Overview** \| **Package** \| **Class** \| **Tree** \| **Deprecated** \| **Index** \| **Help** \| **Weka's home** | |  |
| **PREV CLASS**   **NEXT CLASS** | **FRAMES**    **NO FRAMES**     **All Classes** |  |
| SUMMARY: NESTED \| FIELD \| CONSTR \| METHOD | DETAIL: FIELD \| CONSTR \| METHOD |  |


---
